# Dawson's Fingers in Cerebral Small Vessel Disease

**DOI:** 10.3389/fneur.2020.00669

**Published:** 2020-07-24

**Authors:** Aowei Lv, Zaiqiang Zhang, Ying Fu, Yaping Yan, Li Yang, Wenli Zhu

**Affiliations:** ^1^Department of Neurology, Tianjin Neurological Institute, Tianjin Medical University General Hospital, Tianjin, China; ^2^Department of Neurology, China National Clinical Research Center for Neurological Diseases, Beijing Tiantan Hospital, Capital Medical University, Beijing, China; ^3^Central Laboratory, Department of Neurology and Institute of Neurology, The First Affiliated Hospital of Fujian Medical University, Fuzhou, China; ^4^Key Laboratory of the Ministry of Education for Medicinal Resources and Natural Pharmaceutical Chemistry, National Engineering Laboratory for Resource Development of Endangered Crude Drugs in Northwest of China, College of Life Sciences, Shaanxi Normal University, Xi'an, China

**Keywords:** Dawson's fingers, cerebral small vessel disease, multiple sclerosis, diabetes mellitus, magnetic resonance imaging

## Abstract

To explore Dawson's fingers in cerebral small vessel disease (CSVD) and factors related to the development of Dawson's finger, we collected and analyzed clinical data of 65 patients with CSVD. We found a venous abnormality feature called Dawson's fingers around the ventricles in magnetic resonance images (MRIs) of 20 out of 65 patients with CSVD (30. 8%). A significant association between Dawson's fingers and diabetes mellitus (DM) was also detected (30 vs. 8.9%, *P* < 0.05). CSVD patients with Dawson's fingers had significantly increased cerebral microbleeds (CMB) (44.2 vs. 75.0%, *p* < 0.05), lacunae (66.7 vs. 95.0%, *p* < 0.05), and white matter hyperintensity (WMH) (*p* < 0.05) damage, and these patients exhibited significant cognitive domain impairment as assessed via Montreal Cognitive Assessment (MoCA) (18.9 ± 1.8 vs. 24.0 ± 0.8, *p* < 0.05) and Mini-Mental State Examination (MMSE) (24.5 ± 1.1 vs. 26.6 ± 0.6, *p* < 0.05). Our results show a distinctly high incidence of Dawson's fingers in CSVD patients and identify a significant association with DM, thus yielding insights about the appropriate use of Dawson's fingers, a venous imaging marker, to explore the basic pathophysiology of CSVD.

## Introduction

Cerebral small vessel disease (CSVD) refers to a broad class of cerebrovascular diseases that affect the small arterioles, venules, and capillaries with a variety of different causes (1, 2). Age-related and hypertension-related CSVD are the most common forms (3, 4). Despite its diverse pathogenesis modes, the consequences of CSVD on brain parenchyma elicit similar neuroimaging markers, including lacunes, white matter hyperintensity (WMH), enlarged perivascular space (EPVS), and cerebral microbleeds (CMB) (2). In general, small vessels cannot be visualized in computed tomography angiography (CTA) or magnetic resonance angiography (MRA), but the corresponding parenchymal lesions of small vessels can be captured on magnetic resonance imaging (MRI). The STandards for Reporting Vascular changes on nEuroimaging (STRIVE) is an international neuroimaging standard that classifies and defines CSVD markers (5). For the diagnosis of CSVD, MRI is more specific than clinical criteria. The characteristics of neuroimaging markers are indicative of etiological analysis (5). Notably, arteriolosclerosis is associated with the pathogenesis of sporadic CSVD, yet relatively little attention has been focused on potential roles of venules in CSVD. Thus, the role of venules in the pathogenesis of CSVD, as well as the potential for use of venule abnormalities as imaging markers for diagnosing CSVD, remain unclear.

Central vein sign (CVS), a vein positioned centrally in brain white matter lesions (WMLs), has been considered to be an imaging marker for multiple sclerosis (MS) (6–8), a chronic demyelination and neurodegenerative disease caused by central nervous system (CNS) inflammation. Some studies show that CVS can distinguish MS from mimic diseases (9, 10) including CSVD (11–16). Dawson's fingers are elongated, flame-shaped lesions perpendicular to the lateral ventricle wall on fluid attenuated inversion recovery (FLAIR)/T2 weighted images. T2-weighted/FLAIR MRI allows visibility of venule within WMLs, thus representing inflammatory activity surrounding venule (17). It makes sense that Dawson's fingers are widely used as important imaging markers for the diagnosis and differential diagnosis of MS (18, 19). Complicating matters, CSVD shares some imaging features with MS, including white matter demyelination and brain atrophy. Demyelination around the ventricle is a useful imaging marker for distinguishing MS from other diseases (18). Dawson's fingers, indicating perivascular inflammation around veins and venules, has close pathological association with CVS. Previous studies showed Dawson's fingers can distinguish MS from neuromyelitis optica spectrum disorder (18, 20) and MOG antibody disease (20), but the condition of Dawson's fingers in CSVD is still unknown.

This study aimed to (1) determine the proportion of Dawson's fingers in CSVD; (2) explore the factors contributing to Dawson's fingers in patients with CSVD; (3) determine the imaging and clinical characteristic of CSVD patients with Dawson's fingers; and (4) explore if Dawson's fingers can be used as a specific sign to distinguish MS from CSVD.

## Methods

### Patients and Population

This was a prospective observational study. Patients were consecutively recruited from Tianjin Medical University General Hospital and Beijing Tiantan Hospital. Only patients with clinically manifested CSVD and at least one of the imaging features (lacuna, WMH, EPVS, and CMBs) were eligible to participate in this study. MS was diagnosed using 2017 McDonald criteria. Main exclusion criteria were patients with (1) unidentified vascular risk factors for CSVD such as a history of hypertension, hypercholesterolemia, diabetes mellitus (DM), smoking, and alcohol consumption; (2) evidence of other diseases that could potentially also cause WMLs; and (3) contraindications to MRI scanning.

### Standard Protocol Approvals, Registrations, and Patient Consents

The Ethics Committees of Tianjin Medical University and Beijing Tiantan Hospital approved this study. We obtained informed consent from all the participants.

### Image Acquisition Protocol

All brain MRI was performed on a 3.0-T scanner (Magnetom Trio Tim; Siemens). The routing sequences included T2, FLAIR, T1, diffusion-weighted imaging (DWI), and susceptibility-weighted imaging (SWI) (Supplemental Table 1). All MRIs were assessed for the presence, location, and size of lesions (WMH, enlarged perivascular space [EPVS], lacunes, microbleeds) according to the STandards for Reporting Vascular Changes on nEuroimaging (STRIVE) recommendations. The total SVD score (range 0–4) was calculated according to the individual imaging features by awarding points as follows: 1 for any lacuna, 1 for any microbleed, 1 for moderate-to-severe EPVS in the basal ganglia (EPVS >10), and 1 for WMHs (deep tissue: Fazekas score 2 or 3 and/or periventricular: Fazekas score 3). Individual WM lesion masks were manually delineated based on their T2 images using MRIcro software (http://www.cabiatl.com/mricro/mricro/). WMH lesion volume was calculated within the masks.

Dawson's finger, an elongated, flame-shaped WMH lesion perpendicular to the lateral ventricle wall, was here defined based on T2/FLAIR images. All assessments were executed independently by two trained neurologists who were blinded to the clinical details. The interclass correlation coefficient was 0.86. If there was a discrepancy between the two raters, assessment from a third rater was used.

### Clinical Assessment

Each patient underwent clinical assessment by two neurologists. The demographic factors, clinical factors, and vascular risk factors evaluated included the following: age, sex, hypertension [systolic blood pressure (SBP) >140 mm Hg or diastolic blood pressure (DBP) >90 mmHg], DM (plasma glucose 2 h after a meal ≥11.1 mmol/L or fasting plasma glucose ≥ 7.0 mmol/L), hyperlipidemia (triglycerides >1.7 mmol/L or serum total cholesterol level >5.72 mmol/L), Modified Rankin Scale (mRS), and disease duration, current smoking (21). Laboratory examination results also included atrial fibrillation, as well as assessment of cognition [Montreal Cognitive Assessment (MoCA) and Mini-mental State Examination (MMSE)] of the CSVD patients. All clinical and laboratory factors were fully assessed in all participants.

### Statistical Analysis

Continuous variables with normal distributions, such as age and lesion volumes, are presented as the mean ± standard error of mean. All continuous variables were compared using the Student's *t*-tests for independent samples. Those with non-normal distributions are presented as medians, and interquartile range (IQR) for non-parametric data are quoted. In the univariable analyses, we used χ^2^-tests for lacunes, CMBs, WMH, and EPVS (Fisher's exact test when the expected value was <5) between patients with or without Dawson's fingers. For variables that were non-normally distributed, Mann-Whitney or the Kruskal-Wallis tests were used, as appropriate, or the data were log transformed for parametric testing. ROC curves were used to evaluate the diagnostic value of Dawson's fingers. Statistical Product and Service Solutions (SPSS) for Windows version 17.0 software (Chicago, IL, USA) was used for the analyses. Values of *p* < 0.05 were considered statistically significant.

### Data Availability

Data supporting the findings of this study are available from the corresponding author upon request.

## Result

### Dawson's Fingers Appear in Patients With CSVD

**Figure 1:**
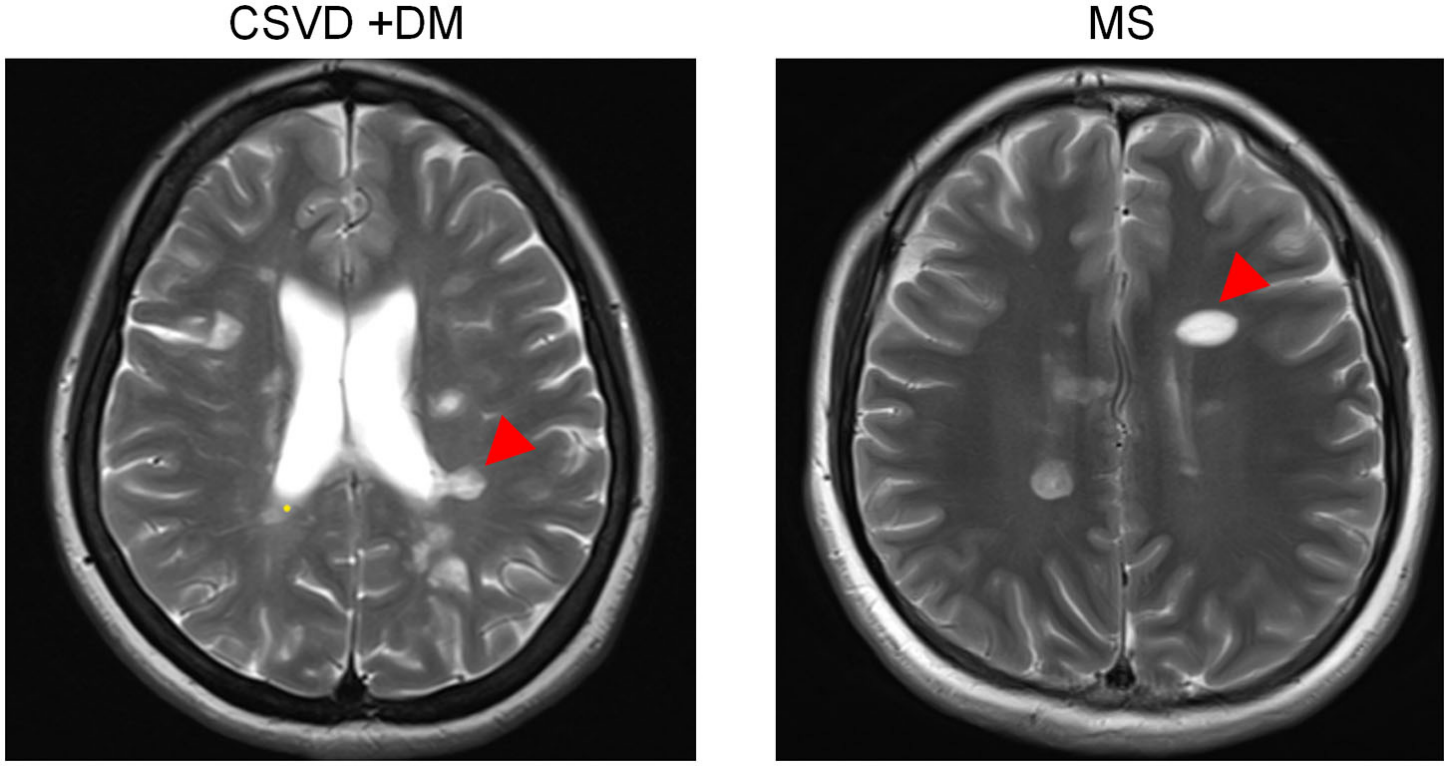
Dawson's fingers of cerebral small vessel disease (CSVD) patients with diabetes mellitus (DM). The venous abnormality feature Dawson's fingers around the ventricles in the fluid attenuated inversion recovery (FLAIR) of both multiple sclerosis (MS) and CSVD patients (arrowheads). Dawson's fingers can be used as a neuroimaging marker of MS and can distinguish between CSVD and MS, but the utility of this marker decreased in patients with DM.

Most often, CSVD is considered to be an arterial-related disease based only on radiological phenotypes; signs of CSVD on conventional MRI markers include CMB, lacunes, WMH, and moderate to severe EPVS (5). However, minimal attention has been paid to venous abnormalities in patients with CSVD. To verify the condition of venous vessels in patients with CSVD, we assessed the appearance of Dawson's fingers among 65 patients with CSVD. The baseline characteristics of the CSVD and MS groups are displayed in Table 1. We found a venous abnormality feature identified as Dawson's fingers around the ventricles in the MRI of CSVD patients (Figure 1). Dawson's fingers were present in 20 of 65 patients with CSVD (30.8%). There were, however, more patients with MS (32/46) than CSVD (20/65) that had Dawson's fingers (69.6 vs. 30.8%, *P* < 0.001); thus, Dawson's fingers can still be used as a neuroimaging marker to distinguish between CSVD and MS. The area under the ROC curve for Dawson's fingers was 0.694 (*P* = 0.001) (sensitivity 71% and specificity 69%).

**Table 1:** Demographic and clinical characteristics of patients with MS and CSVD.

**Characteristic**	**MS (*N* = 46)**	**CSVD (*N* = 65)**	***P*-value**
Age, year, median (IQR)	31.5 (24.8–36.3)	54 (47–61)	0.000
Male sex, *n* (%)	15 (32.6)	31 (47.7)	0.112
Hypertension, *n* (%)	1 (2.2)	43 (66.2)	0.000
SBP, mmHg, median (IQR)	115 (108–120)	134 (122–144)	0.000
DBP, mmHg, median (IQR)	75 (70–80)	80 (73.5–86.5)	0.000
Hyperlipidemia, *n* (%)	11 (23.9)	29 (44.6)	0.025
Diabetes, *n* (%)	1 (2.2)	10 (15.4)	0.049^&^
Atrial fibrillation (%)	0 (0)	1 (1.6)	1.000
Current smoking, *n* (%)	1 (2.2)	22 (33.8)	0.000
Alcohol, *n* (%)	1 (2.2)	17 (26.2)	0.001
Disease duration, year, median (IQR)	5 (1.9–7.2)	2 (0.6–4.0)	0.001

*Hypertension = Systolic blood pressure >140 mmHg and/or diastolic blood pressure >90 mmHg; Diabetes = Fasting plasma glucose ≥7.0 millimoles per liter and/or plasma glucose after a meal for 2 h ≥11.1 millimoles per liter; Hyperlipidemia = Serum total cholesterol levels >5.72 millimoles per liter; and/or triglycerides >1.7 millimoles per liter*.

*MS, multiple sclerosis; CSVD, cerebral small vessel disease; IQR, interquartile range; SBP, systolic blood pressure; DBP, diastolic blood pressure; & P value < 0.05*.

### Demographics and Vascular Risk Factors in Patients With and Without Dawson's Fingers

To explore the factors contributing to Dawson's fingers in CSVD, we performed demographic and vascular risk factor analysis of CSVD patients with and without Dawson's fingers. With the exception of patients with DM, these analyses did not identify differences between CSVD patients with Dawson's fingers compared to patients without Dawson's fingers in demographic factors, number of attacks, or disease duration, or with other vascular risk factors (e.g., hypertension, hyperlipidemia). There was a statistically significant difference between DM status and CSVD patients with (6/20) or without (4/45) Dawson's fingers (30 vs. 8.9%, *P* < 0.05, Table 2). The utility of Dawson's fingers to distinguish between CSVD from MS (32/46) decreased in CSVD patients with DM (6/10) (AUC = 0.454; *P* > 0.05).

**Table 2:** Demographic and clinical characteristics of CSVD patients with or without Dawson's fingers.

**Characteristic**	**CSVD without Dawson's fingers (*N* = 45)**	**CSVD with Dawson's fingers (*N* = 20)**	***P*-value**
Age, year (mean, SE)	54.0 ± 1.8	52.5 ± 2.6	0.378
Male sex, *n* (%)	18 (40.0)	13 (65.0)	0.063
Hypertension, *n* (%)	32 (71.1)	11 (55.0)	0.205
SBP, mmHg, median (IQR)	133 (122–144)	137 (121–146)	0.754
DBP, mmHg, median (IQR)	80 (74.5–85.0)	80 (70.0-88.0)	0.938
Hyperlipidemia, *n* (%)	29 (42.2)	10 (50.0)	0.560
Diabetes mellitus, *n* (%)	4 (8.9)	6 (30.0)	0.029
Current smoking, *n* (%)	14 (31.3)	8 (40.0)	0.485
Alcohol, *n* (%)	12 (26.7)	5 (25.0)	0.888
Disease duration, year (median, range)	2.0 (0.2–20.0)	2.5 (0.2–8.0)	0.209
mRS (mean, median, range)	1.2, 1 (1–4)	1.3, 1 (1–3)	0.244
EDSS (median, range)	2 (2–8)	3 (2–6)	0.147
Number of attacks^*^ (median, range)	1 (0–4)	1 (0–4)	0.630
Atrial fibrillation (%)	1 (2.4)	0 (0)	1.000

*Hypertension = Systolic blood pressure >140 mmHg and/or diastolic blood pressure >90 mmHg; Diabetes = Fasting plasma glucose ≥7.0 millimoles per liter and/or plasma glucose after a meal for 2 h ≥11.1 millimoles per liter; Hyperlipidemia = Serum total cholesterol levels >5.72 millimoles per liter; and/or triglycerides >1.7 millimoles per liter*.

* *Only note ischemic attack in CSVD, but not include transient ischemic attack (TIA)*.

*CSVD, cerebral small vessel disease; SE, standard error; SBP, systolic blood pressure; DBP, diastolic blood pressure; IQR, interquartile range; mRS, modified Rankin scale, range from 0 to 6; EDSS, Expanded Disability Status Scale, range from 0 to 10, higher scores indicate a greater degree of disability*.

### Dawson's Fingers Affect Cognition and Imaging in Patients With CSVD

**Figure 2:**
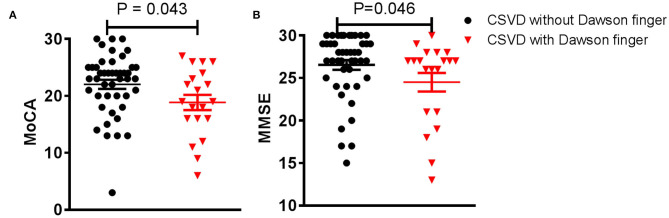
Dawson's fingers are associated with cognitive impairment in small vessel disease (CSVD) patients. **(A,B)** Montreal Cognitive Assessment (MoCA) and Mini-mental State Examination (MMSE) scores of CSVD patients with Dawson's fingers were lower compared to those without Dawson's fingers (*p* < 0.05). The data are presented as the mean ± standard error of mean (SEM).

Dawson's fingers appear in patients with CSVD and are related to DM. To observe the impact of Dawson's fingers on clinical function and imaging of CSVD patients, we carefully analyzed imaging for all patients. WMH, lacunes, and CMB have a statistically significant difference between CSVD with and without Dawson's fingers, especially mixed lacunes (*p* < 0.001, Table 3). Then, we analyzed the function of CSVD with and without Dawson's fingers. No differences in mRS (1.3 ± 0.1 vs. 1.2 ± 0.1, *p* = 0.244) were observed, however, MoCA (18.9 ± 1.8 vs. 24.0 ± 0.8, *p* < 0.05) and MMSE (24.5 ± 1.1 vs. 26.6 ± 0.6, *p* < 0.05) were significantly different between CSVD with and without Dawson's fingers (Figure 2). Therefore, the cognitive level of CSVD patients with Dawson's fingers is significantly decreased compared to CSVD patients where Dawson's fingers were not detected.

**Table 3:** Cerebral small vessel disease markers of patients with or without Dawson's fingers.

**Characteristic**	**CSVD without Dawson's fingers (*N* = 45)**	**CSVD with Dawson's fingers (*N* = 20)**	***P*-value**
Cerebral Microbleeds, *n* (%)	19 (42.2)	15 (75.0)	0.018
Lobar CMBs	18	13	0.105
Deep CMBs	16	14	0.015
Mixed CMBs	12	12	0.014
CMB burden			0.011
0 (0 CMB)	26	5	0.017
1 (1–5 CMB)	5	3	0.693
2 (6–15 CMBs)	6	10	0.004
3 (>15 CMBs)	7	2	0.710
Lacunes, *n* (%)	30 (66.7)	19 (95.0)	0.014
Lobar lacunes	24	17	0.015
Deep lacunes	21	18	0.001
Mixed lacunes	14	16	<0.001
Lacunes burden			0.003
0 (0 lacunes)	15	2	0.067
1 (1–3 lacunes)	14	2	0.117
2 (4–10 lacunes)	12	8	0.383
3 (>10 lacunes)	4	8	0.005
PVWMH (Fazekas score), *n* (%)			0.050
0–1	21	3	0.025
2	8	6	0.332
3	16	11	0.178
DWMH (Fazekas score), *n* (%)			0.054
0–1	28	6	0.030
2	9	8	0.127
3	8	6	0.332
Total WMH severity (Fazekas score), *n* (%)			0.027
0–2	19	2	0.011
3–4	16	9	0.583
5–6	10	9	0.080
WMH volume, ml (median, IQR)	17 (10–65)	38 (18–75)	0.153
Severe CSO-EPVS, *n* (%)	9	6	0.524
Severe BG-EPVS, *n* (%)	14	10	0.171
Total SVD score (median, IQR)	3 (1.5–3)	3 (3–4)	0.005

*CMBs, cerebral microbleeds; CSO, centrum semiovale; BG, basal ganglia; EPVS, enlarged perivascular spaces; PVWMH, periventricular white matter hyperintensities; DMWH, deep white matter hyperintensities; CSVD, cerebral small vessel disease; IQR, Inter Quartile Range*.

*The CMBs were divided into lobar CMBs group, deep CMBs group, and mixed CMBs group on the basement of locations (22). CMBs in cortices or subcortical and periventricular white matter were lobar CMBs. Deep CMBs were in areas including deep white matter (corpus callosum, internal, external, and extreme capsule), deep nuclei (basal ganglia and thalamus), and infratentorial structures (brain stem and cerebellum). Mixed CMBs included both lobar and deep CMB. The scoring standard of CMB burden is as follows: 0 points for 0 CMB, 1 point for 1–5 CMBs, 2 points for 6–15 CMBs, 3 points for over 15 CMBs*.

*Lacunes were defined as a subcortical cavity filled with fluid having a similar signal to CSF, shaped round or ovoid, ranging from 3 to 15 mm in diameter and they were classified based on T1, T2, FLAIR images (5). Lacunes were classified in three groups: lobar lacunes (in centrum semiovale, frontal, parietal, insular/subinsular, temporal, and occipital lobes), deep lacunes (in thalamus, basal ganglia, caudate, internal, and external capsule), and mixed lacunes (including both lobar and deep) (23). The scoring standard of lacunes burden is as follows: 0 points for 0 lacune, 1 point for 1–3 lacunes, 2 points for 4–10 lacunes, 3 points for over 10 lacunes*.

*WMH were defined as hyperintense of variable size in the demyelination of white matter on T2/FLAIR images on MRI. Fazekas grading scale was used to assess the level of WMH*.

*Severe BG-EPVS or CSO-EPVS were defined as >20 in the basal ganglia or centrum semiovale*.

*x^2^-test and Mann–Whitney test to test for differences between patient with Dawson's fingers*.

## Discussion

CSVD mainly refers to a group of diseases in which arterioles are damaged; whether there is venous injury has been controversial. Dawson's fingers are an important imaging marker in the diagnosis of MS. To determine whether there is a venous abnormality in CSVD, we collected and analyzed the clinical data of small arteriosclerotic CSVD and found a venous abnormality feature (Dawson's fingers) around the ventricles in the MRI of 30.8% of patients. Statistical analysis revealed an association between Dawson's fingers and DM in the CSVD patients of our cohort. Moreover, we found that Dawson's fingers are associated with decreased cognitive ability.

CSVD is typically considered to be a small artery system-related disease (24). The most prevalent etiological type for sporadic CSVD is arteriolosclerosis caused by aging or vascular risk factors (1, 25). The pathological feature of arteriosclerosis is narrowing of the lumen and thickening of the vessel wall, and atherosclerosis has a strong relationship with the clinical symptoms of CSVD such as subsequent cognitive decline and neuroimaging features, e.g., CMB (26). Further, previous work has established that high blood pressure is as an independent risk factor for CSVD (27). It is notable that relatively few studies of CSVD have focused on veins. CVS, as a venous sign, is a common imaging feature identified with MS, which is only present in 6–7% proportion of CSVD patients. However, Dawson's fingers, another venous sign, were present in more than 30% of patients with CSVD in this study. These results clearly indicate that venous problems can occur amongst CSVD patients, thus supporting an important role for venous problems in the pathogenesis of CSVD.

The pathogenesis of Dawson's fingers corresponds to the scope of perivenous inflammation, which is perpendicular to lateral ventricles along subependymal veins (28). As noted, Dawson's fingers can be used as an imaging marker in differential diagnosis of MS from its mimics. Additionally, it has been reported that Dawson's fingers can also help differentiate other autoimmune demyelinating diseases from MS (18, 29, 30). However, a common challenge in the clinic is difficulties in distinguishing MS from CSVD on the basis of MRI appearance. For example, in middle-aged or elderly patients, CSVD has a high prevalence of leukoencephalopathy (31), so a variety of imaging markers are used to distinguish MS from CSVD. In our study, 20 of 65 CSVD patients (30.8%) had Dawson's fingers, whereas 32 of 46 MS patients (69.6%) had Dawson's fingers, and the difference in proportions between MS and CSVD groups was significant. Thus, despite its apparently abnormally high prevalence in CSVD patients in our cohort, Dawson's fingers can still be used to distinguish between MS and CSVD. However, our data also shows that the utility of this marker decreased in patients with DM. Therefore, careful consideration of this and other imaging markers for the differential diagnosis of MS and CSVD, and especially in patients with DM, merits attention.

DM can contribute to the development of CSVD by initiating and accelerating the development of arteriolosclerosis (26). It is known that CSVD patients with DM exhibit CMB, WMH, and lacunes more commonly than do CSVD patients without DM (32). It is also long been known that DM can cause venous damage (33). Our study showed significantly higher prevalence of Dawson's fingers in CSVD patients with DM. This result suggested that DM may be associated with the occurrence of venous problems in CSVD, but this needs to be confirmed in a large longitudinal study.

Our study has some limitations, including its nature as an explorative study with a relatively small patient cohort. Therefore, we encourage researchers to use caution in directly extrapolating our results to other research; future studies should include additional participants. In addition, this study did not conduct any follow-up scientific explorations of the potential pathogenic mechanism of Dawson's fingers in CSVD patients. We did rigorously evaluate the clinical data of all CSVD patients. Our work also therefore suggests that future examinations of the pathophysiology of CSVD should pay attention to venous abnormalities and should certainly consider potential impacts from diabetes.

## Data Availability Statement

All datasets generated for this study are included in the article/Supplementary Material.

## Ethics Statement

The studies involving human participants were reviewed and approved by the Ethics Committees of Tianjin Medical University and Beijing Tiantan Hospital. Written informed consent to participate in this study was provided by the participants' legal guardian/next of kin.

## Author Contributions

YF conceptualized the study, acquired funding for this study, and designed the study. YF, ZZ, and LY collected the data. LY, YY, WZ, and AL analyzed the data. YF, WZ, and AL wrote the manuscript. All authors contributed to the article and approved the submitted version.

## Conflict of Interest

The authors declare that the research was conducted in the absence of any commercial or financial relationships that could be construed as a potential conflict of interest.
