# Supplementary material for: Dawson's Fingers in Cerebral Small Vessel Disease
Source: Front Neurol. 2020 Jul 24;11:669. doi: 10.3389/fneur.2020.00669 (PMC7396560; doi:10.3389/fneur.2020.00669)
Supplement: Supplementary file 1 [file Table_1.DOCX]

| **Supplemental Table 1. Magnetic resonance imaging parameters** | | | | | | |
| --- | --- | --- | --- | --- | --- | --- |
| **Sequence** | **TR**  **(ms)** | **TE**  **(ms)** | **Resolution**  **(mm)** | **Thick slices**  **(mm)** | **Interslice gap**  **(mm)** | **Additional Details** |
| T2_2D | 4500 | 84 | 0.6 x 0.6 | 5 | 1.5 | Echo train length =17 |
| FLAIR_2D | 8000 | 94 | 0.9 x 0.9 | 5 | 1.5 | TI = 2,250 ms |
| T1_2D | 1200 | 11 | 0.4 x 0.4 | 5 | 1.5 | TI = 800 ms |
| DWI_2D | 4200 | 93 | 1.2 x 1.2 | 5 | 1.5 | b = 0 and 1000 s/mm^2^ |
| SWI_3D | 27 | 20 | 0.9 x 0.9 | 2.5 | 0 | Flip angle =15° |
| TE = echo time; TI= inversion time; TR= repetition time. | | | | | | |
